# Simultaneous saccharification and lactic acid fermentation of the cellulosic fraction of municipal solid waste using *Bacillus smithii*

**DOI:** 10.1007/s10529-020-03049-y

**Published:** 2020-11-21

**Authors:** Micaela G. Chacón, Christopher Ibenegbu, David J. Leak

**Affiliations:** Department of Biology and Biochemistry, University of Bath, Bath, BA2 7AY England, UK

**Keywords:** *Bacillus smithii*, Lactic acid, Municipal solid waste, Simultaneous saccharification and fermentation

## Abstract

**Objective:**

A primary drawback to simultaneous saccharification and fermentation (SSF) processes is the incompatibility of the temperature and pH optima for the hydrolysis and fermentation steps—with the former working best at 50–55 °C and pH 4.5–5.5. Here, nine thermophilic *Bacillus* and *Parageobacillus* spp. were evaluated for growth and lactic acid fermentation at high temperature and low pH. The most promising candidate was then carried forward to demonstrate SSF using the cellulosic fraction from municipal solid waste (MSW) as a feedstock.

**Results:**

*B. smithii* SA8Eth was identified as the most promising candidate and in a batch SSF maintained at 55 °C and pH 5.0, using a cellulase dose of 5 FPU/g glucan, it produced 5.1 g/L lactic acid from 2% (w/v) MSW cellulosic pulp in TSB media.

**Conclusion:**

This work has both scientific and industrial relevance, as it evaluates a number of previously untrialled bacterial hosts for their compatibility with lignocellulosic SSF for lactic acid production and successfully identifies *B. smithii* as a potential candidate for such a process.

**Electronic supplementary material:**

The online version of this article (10.1007/s10529-020-03049-y) contains supplementary material, which is available to authorized users.

## Introduction

With growing concern over the environmental and socioeconomic impact of using the carbohydrate derived from edible crops as a substrate for the microbial production of energy and biochemicals, focus has shifted towards the use of lignocellulosic feedstocks. One of the cheapest and most abundantly available forms of lignocellulose is the cellulosic fraction recovered from sorting municipal solid waste (MSW), which is produced in significant quantities worldwide (Jensen et al. 2010). While variable by region, the carbohydrate fraction of this material ranges between 30 and 69%, making it an attractive fermentation feedstock (López-Gómez et al. 2019). Recently, several research groups have demonstrated the use of MSW derived carbohydrates for the production of short chain alcohols and acids (Nwobi et al. 2015; Probst et al. 2015; Farmanbordar et al. 2018; López-Gómez et al. 2019).

Lactic acid is a widely used platform chemical in the food, pharmaceutical, chemical, and textile industries, and has recently become an important building block for the production of the bioplastic polylactic acid (PLA) (Aulitto et al. 2019). Industrially, it is typically produced via fermentation with lactic acid bacteria using purified carbohydrate-based feedstocks, making the process costly and increasingly unsustainable (Van Der Pol et al. 2016). This has stimulated research targeting reduction in production costs, including improved purification methods and developing processes that utilize less-expensive lignocellulose-based substrates (Van Der Pol et al. 2016; Takano and Hoshino 2016; Zhou et al. 2016; Chen et al. 2017). Most process concepts for using lignocellulose begin with a thermo-chemical pretreatment of the feedstock (pretreatment), followed by an enzymatic saccharification of the cellulose and hemicellulose fractions, producing monomeric sugars for microbial fermentation. In separate hydrolysis and fermentation (SHF) these steps are carried out sequentially, but it is also feasible to do both steps together in a process called simultaneous saccharification and fermentation (SSF) (Olofsson et al. 2008). SSF offers some advantages over SHF, as hydrolytic enzyme end-product inhibition is alleviated as the liberated carbohydrates are rapidly consumed by the microorganism. Further, use of a single vessel reduces equipment costs, shortens the process time, and has been shown to result in higher production rates (Berlowska et al. 2016). However, a significant disadvantage to SSF is that the optimal operational temperature and pH of the hydrolytic enzymes used for saccharification (50–55 °C and pH 4.5–5.5) are incompatible with the growth and fermentation optima of most microorganisms (Aulitto et al. 2019). For this reason, SSF is usually carried out at the optimal growth temperature of the industrial microbe, which is typically mesophilic, thus resulting in the need for higher enzyme loads to compensate for the less than optimal enzyme activity (Stenberg et al. 2000; Sreenath et al. 2001; Yáñez et al. 2003; John et al. 2009; Berlowska et al. 2016; Takano et al. 2016; Cheng et al. 2018; Dhandapani et al. 2019). More recently, however, the development of SSF processes that utilize moderate thermophiles capable of growth within the 50–55 °C range has been of interest, with *Bacillus coagulans* and thermophilic *Lactobacillus* sp. showing promise for lactic acid production (Patel et al. 2006; Hu et al. 2015; Van Der Pol et al. 2016; Zhou et al. 2016; Pleissner et al. 2017; Aulitto et al. 2019; Jiang et al. 2019; López-Gómez et al. 2019). The use of a thermophilic host offers additional process benefits including (i) reduced contamination risk from mesophiles, (ii) increased substrate and product solubility, and (iii) reduced cooling costs compared to mesophilic fermentations (Bosma et al. 2015). For this reason, in this study we have extended the portfolio of thermophilic microbial platforms compatible with SSF which are potentially useful for industrial lactic acid production.

Based on an initial screen (catabolic potential, growth pH, temperature and lactic acid production) of our culture collection, four strains of *Bacillus caldolyticus*, four of *Parageobacillus caldoxylosyliticus*, and one of *Bacillus smithii* were selected for further evaluation. The most promising candidate was then carried forward to demonstrate SSF using the un-hydrolysed cellulosic fraction of MSW as the feedstock. Although the production of lactic acid by fermentation of hydrolysed MSW pulp has been described previously (López-Gómez et al. 2019, 2020) to our knowledge this is the first example in which SSF has been employed.

## Material and methods

### Municipal solid waste (MSW)

MSW cellulosic pulp was provided by Fiberight Ltd. from its pilot plant in Lawrenceville (Virginia, USA). Plastics and metals were removed, and the biodegradable fraction was pulped using a hydrothermal process. The resulting material had a total solids (TS) content of 33%, and a solids composition of: 55% glucan, 25% lignin, 11% xylan, 6% arabinan/galactan/mannan, and 3% ash. MSW pulp was autoclaved at 121 °C for 15 min prior to experimental use. MSW sugar-rich hydrolysate was also provided by Fiberight Ltd from its pilot plant production facility in Southampton (Hampshire, UK).

### Microorganisms

A selection of nine thermophilic/moderately thermophilic bacterial species were selected from an in-house culture collection for screening: four *B. caldolyticus* strains 00,275, 00,452, 12,042, and 12,028; four *P. caldoxylosyliticus* strains 10,020, 10,087, 10,092, and 10,146; and one *B. smithii* strain SA8Eth (internal reference numbers, source/provider listed in Supplementary Table 1). Each strain was gradually adapted to an environmental pH of 5.5 by serial passaging. Initial culturing was carried out in Tryptic Soy Broth (TSB; 17 g/L tryptone, 3 g/L soy peptone, 5 g/L NaCl, 2.5 g/L K_2_HPO_4_, 2.5 g/L glucose) and 50 mM citrate–phosphate buffer adjusted to pH 7.0, followed by TSB adjusted to pH 6.5, then pH 6, and finally pH 5.5. All incubations were carried out for 24 h at 55 °C in flask at 200 rpm. Strains adjusted to pH 5.5 were maintained on TSA (pH 5.5) plates at 4 °C before use.

**Table 1:** Effect of C-Tec2 cellulase cocktail dosage on the initial rate of glucose and xylose release from 2% (DW) MSW pulp at pH 5.0 and 55 °C

C-Tec2 (FPU/g glucan)	Rate of glucose release (mg/L/min)	Rate of Xylose release (mg/L/min)
10	20 ± 0.9	1.4 ± 0.1
5	13.3 ± 0.3	1.2 ± 0.06
2.5	7 ± 0.4	0.8 ± 0.05
1.25	4.3 ± 0.3	0.5 ± 0.08
0.625	3.4 ± 0.1	0.3 ± 0.01

### Growth curves

A starter culture of each strain was grown overnight and used to inoculate 50 mL of TSB (pH 5.5) + 20 g/L glucose to a starting OD_600_ of 0.1, in triplicate shake flasks, which were incubated at 55 °C and 200 rpm. The OD_600_ was measured approximately every hour for 8.5 h. Growth curves of *B. smthii* SA8Eth in either TSB (pH 5.0) + 20 g/L glucose or TSB (pH 5.0) + 20 g/L MSW hydrolysate sugar were also carried out as described above. For this work, MSW hydrolysate sugar describes only the glucose and xylose present within the hydrolysate, thus 20 g/L MSW hydrolysate sugar equates to 20 g/L total glucose plus xylose.

### Xylose utilization

A starter culture of each strain was grown overnight in TSB and used to inoculate TSB (pH 7.0) + 20 g/L xylose to a starting OD_600_ of 0.1, in triplicate. Cultures were incubated at 55 °C and 200 rpm in baffled flasks for 24 h before a final OD_600_ was recorded and culture supernatant samples were taken to assess xylose consumption.

### Fermentation in flasks

A starter culture of each strain was grown overnight in TSB and used to inoculate 40 mL of TSB (pH 7.0) + 20 g/L glucose to an OD_600_ of 1.5 in a 50 mL bottle before being crimp capped. Fermentations were carried out at 55 °C and 200 rpm for 48 h before culture supernatant samples were taken to assess acid production.

### Fermentation of B. smithii SA8Eth in a bioreactor

For batch fermentation in a bioreactor*, B. smithii* SA8Eth was grown overnight in TSB and used to inoculate 1.5 L of TSB (pH 5.0) + 20 g/L MSW hydrolysate in a BIOSTAT B 2 L bioreactor to an OD_600_ of 0.15. Batch fermentation conditions were: 55 °C, pH 5.0 (maintained by the automatic addition of 5 M KOH), 0.4 L/min air flow rate, 400 rpm agitation rate, and antifoam 204 addition on demand. Supernatant samples were taken periodically to monitor the OD_600_, and HPLC analysis.

For SSF fermentation in the bioreactor, *B. smithii* SA8Eth was grown overnight in TSB and used to inoculate 1.5 L of TSB (pH 5.0) + 2% (w/v) MSW pulp in a BIOSTAT B 2 L Bioreactor to an OD_600_ of 0.15. Directly following this, 5 FPU/g glucan C-TEC2 cellulase mix was added to the bioreactor. SSF fermentation conditions were as described above. Samples were taken for HPLC analysis.

### MSW hydrolysis assay using different C-TEC2 concentrations

Hydrolysis assays were carried out for 200 min in triplicate, using a total reaction volume of 30 mL in 250 mL baffled flasks shaken at 250 rpm. Assay conditions were: 2% (w/v) MSW pulp, 50 mM citrate–phosphate buffer (pH 5.0), and either 0.625, 1.25, 2.5, 5, or 10 FPU/g glucan C-TEC2 (Novozymes, Denmark) incubated at 55 °C, with samples taken periodically to assess glucose and xylose release.

### Quantification of lactic acid and carbohydrates

Glucose and xylose monomers were quantified by ion chromatography (Dionex 5000 + , Thermo Fisher Scientific, USA) using a 4 × 250 mm analytical CarboPac PA1 column. Analytes were separated isocratically at 30 °C using 50 mM NaOH as eluent at a flow rate of 1.0 mL/min.

Lactic acid and acetic acid were quantified by HPLC (Agilent 1200 system) using a 300 × 7.8 mm REZEX ROA organic acid column (Phenomenex, USA). Analytes were separated isocratically at 65 °C in 5 mM H_2_SO_4_ at a flow rate of 0.6 mL/min (Wright et al. 2018).

## Results and discussion

### Strain comparison

This work aimed to expand the catalogue of industrially relevant bacilli that could feasibly be used for lactic acid production by SSF of MSW pulp. Towards this end, four *B. caldolyticus* strains (00275, 00452, 12042, and 12028), four *P. caldoxylosyliticus* strains (10020, 10087, 10092, and 10146), and one *B. smithii* strain (SA8Eth) were investigated as potential candidates. These species were selected as they had previously been characterised as thermophiles capable of growth at and above 50 °C (Bergquist et al. 1987; Ahmad et al. 2000; Bosma et al. 2016). The prerequisites for a successful candidate were that they (i) should be able to grow at the temperature and pH optima of the cellulase enzymes used (ie. 50–55 °C and pH 4.5–5.5), (ii) could utilize both C_6_ and C_5_ sugars, and (iii) produced lactic acid as a primary metabolic product under oxygen-limited conditions.

**Fig. 1:**
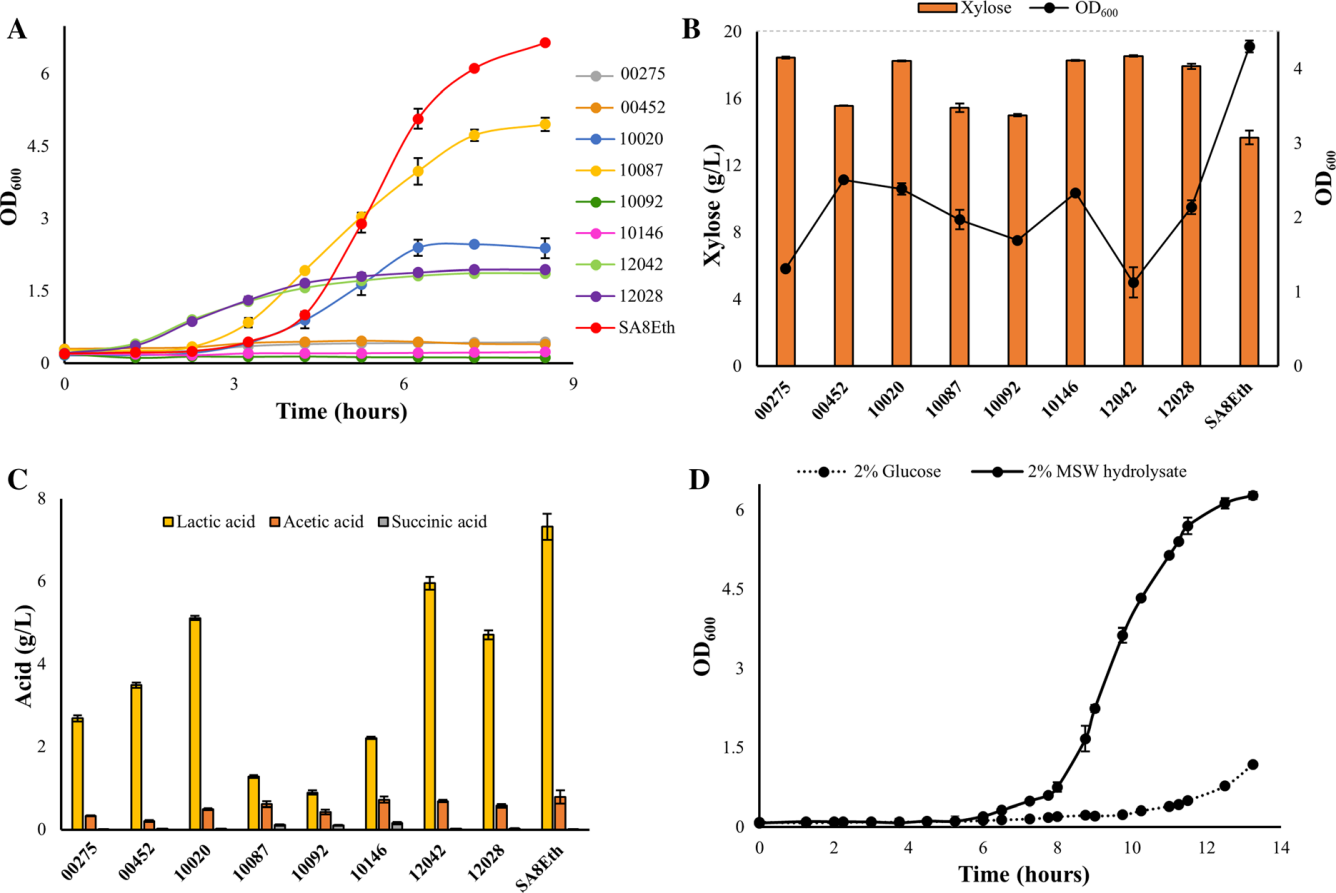
Comparison of nine candidate thermophilic bacterial strains for **a** growth (OD_600_) at 55 °C and pH 5.5, **b** xylose consumption after 24 h, and **c** lactic acid fermentation from glucose after 48 h. *B. smithii* strain SA8Eth was further assessed for **d** growth (OD_600_) at 55 °C and pH 5.0 in either TSB + 20 g/L glucose (dotted line; OD_600_ at 24 h: 2.8 ± 0.17) and TSB + 20 g/L MSW hydrolysate sugar (solid line; OD_600_ at 24 h: 6.6 ± 0.26). Data are the mean ± standard deviation of three biological replicates

Initially, growth curves were generated for each strain at 55 °C in TSB (pH 5.5) + 20 g/L glucose over 8.5 h (Fig. 1a). In repeat experiments, strains 00275, 00452, 10146 and 10092 consistently did not show significant growth over the 8.5 h time course but did by the final OD_600_ reading at 24 h (Supplementary Table 2). An excessively long lag phase is undesirable for an SSF reaction as without microbial growth and metabolism, the associated build-up of monomeric and dimeric sugars released from the lignocellulosic feedstock can result in cellulase enzyme inhibition. The longer fermentation times that result from long lag phases are also undesirable for process economics. These strains, however, did grow well at pH 7.0 (Supplementary Fig. 1). Over the time course at pH 5.5, strains 10087, and SA8Eth both grew well, while 10020, 12028 and 12042 grew moderately well (Fig. 1a).

Next, the ability of each strain to utilize xylose was evaluated, as it accounts for approximately 15–20% of MSW pulp derived sugars (Puri et al. 2013). Each strain was found to be able to utilize xylose when grown in TSB (pH 7.0) + 20 g/L xylose at 55 °C, with most strains reaching an OD_600_ after 24 h that was similar to that when grown under identical conditions in TSB + glucose (Fig. 1b, Supplementary Fig. 2). To evaluate fermentation product profiles, each strain was grown for 48 h under microaerobic conditions at 55 °C in TSB (pH 7.0) + 20 g/L glucose. All strains produced lactic acid as a major product, with minor amounts of acetic acid and succinic acid (Fig. 1c, Supplementary Table 3). The two highest lactic acid producers in shake-flasks were strains 12042 and SA8Eth, resulting in 6.0 g/L and 7.3 g/L, respectively (Fig. 1c). Notably, all of the highest producing strains were able to grow at pH 5.5 and, as the pH of the shake-flask cultures could not be controlled, this suggested that with the exception of strain 10087, the titres reflected the environmental pH at which the cell growth was arrested.

While the pH range for cellulase activity is 4.0–5.5, peak cellulolytic performance was achieved at pH 5.0 for the commercial cocktail used in this work (Farinas et al. 2010; Novozymes application sheet 2017). Therefore, the ability of those strains with the highest lactic acid production (10020, 12028, 12042 and SA8Eth) to grow at pH 5.0 and 55 °C in TSB + 20 g/L glucose was further assessed. Of these, only SA8Eth was able to grow at this initial pH, though it demonstrated an extended lag phase and slow growth, eventually reaching an OD_600_ of 2.8 ± 0.17 at 24 h (Fig. 1d, dashed line). Interestingly, when grown at pH 5.0 in TSB + 20 g/L MSW hydrolysate sugars, SA8Eth exhibited significantly improved growth compared to just glucose supplementation (Fig. 1d, solid line, Supplementary Fig. 4), reaching an OD_600_ of 6.6 ± 0.26 at 24 h. Although it is possible that the hydrolysate was providing additional micro-nutrients it is more likely that it was providing additional buffering capacity to stop the pH dropping below 5.

### *SSF with B. smithii SA8Eth using MSW pulp*

**Fig. 2:**
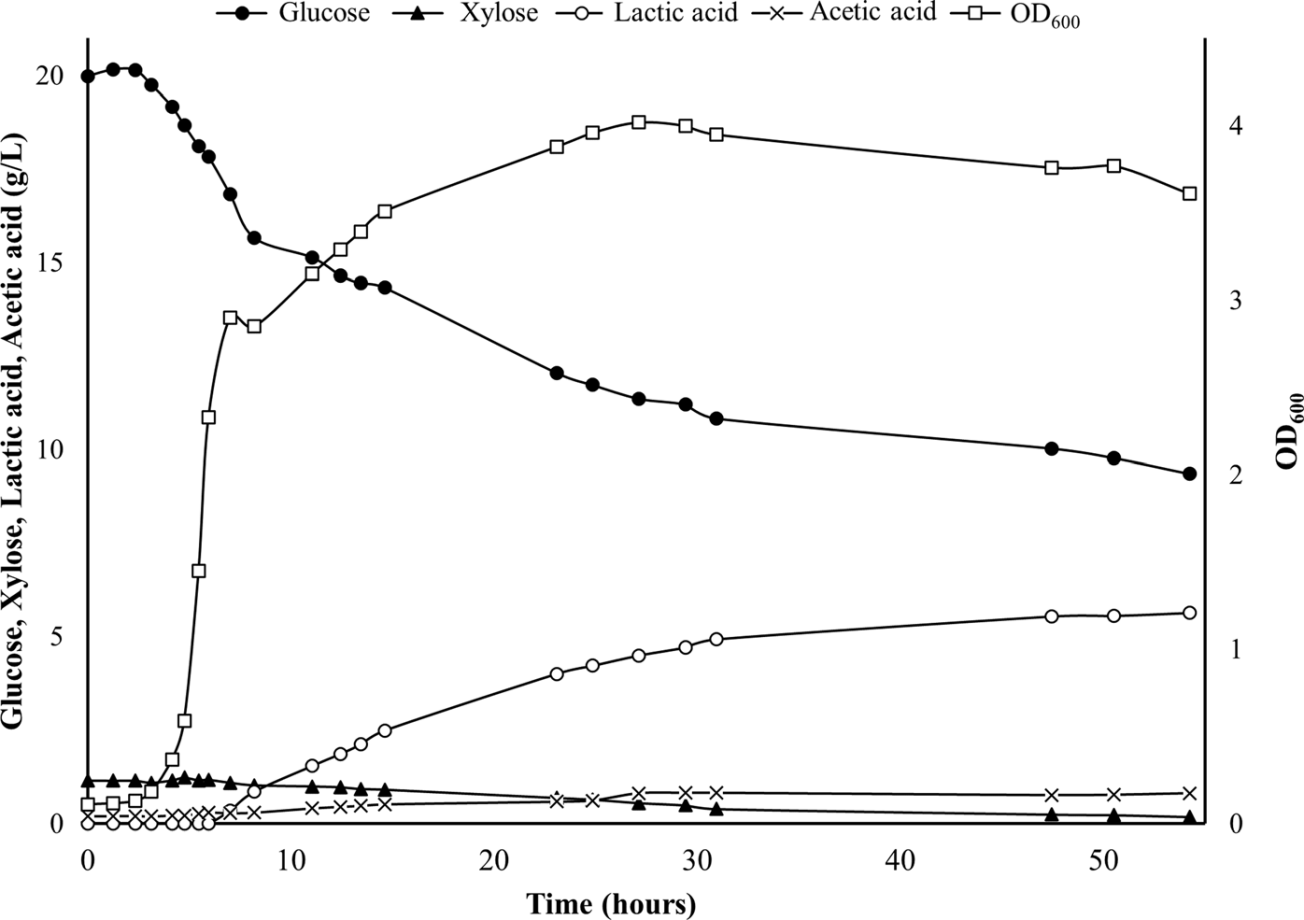
Fermentation of *B. smithii* SA8Eth in TSB + 20 g/L MSW hydrolysate sugar at 55 °C and pH 5.0. Fermentation was performed in a 1.5 L bioreactor. Growth (OD_600_, open squares), glucose/xylose consumption (solid circles and solid triangles, respectively), lactic acid (open circles) and acetic acid (X’s) were tracked over 54 h. Rate of aerobic glucose consumption was calculated between hours 2.33–8.2 h, while glucose consumption during microaerobic fermentation was calculated between hours 15.66–20.15

**Fig. 3:**
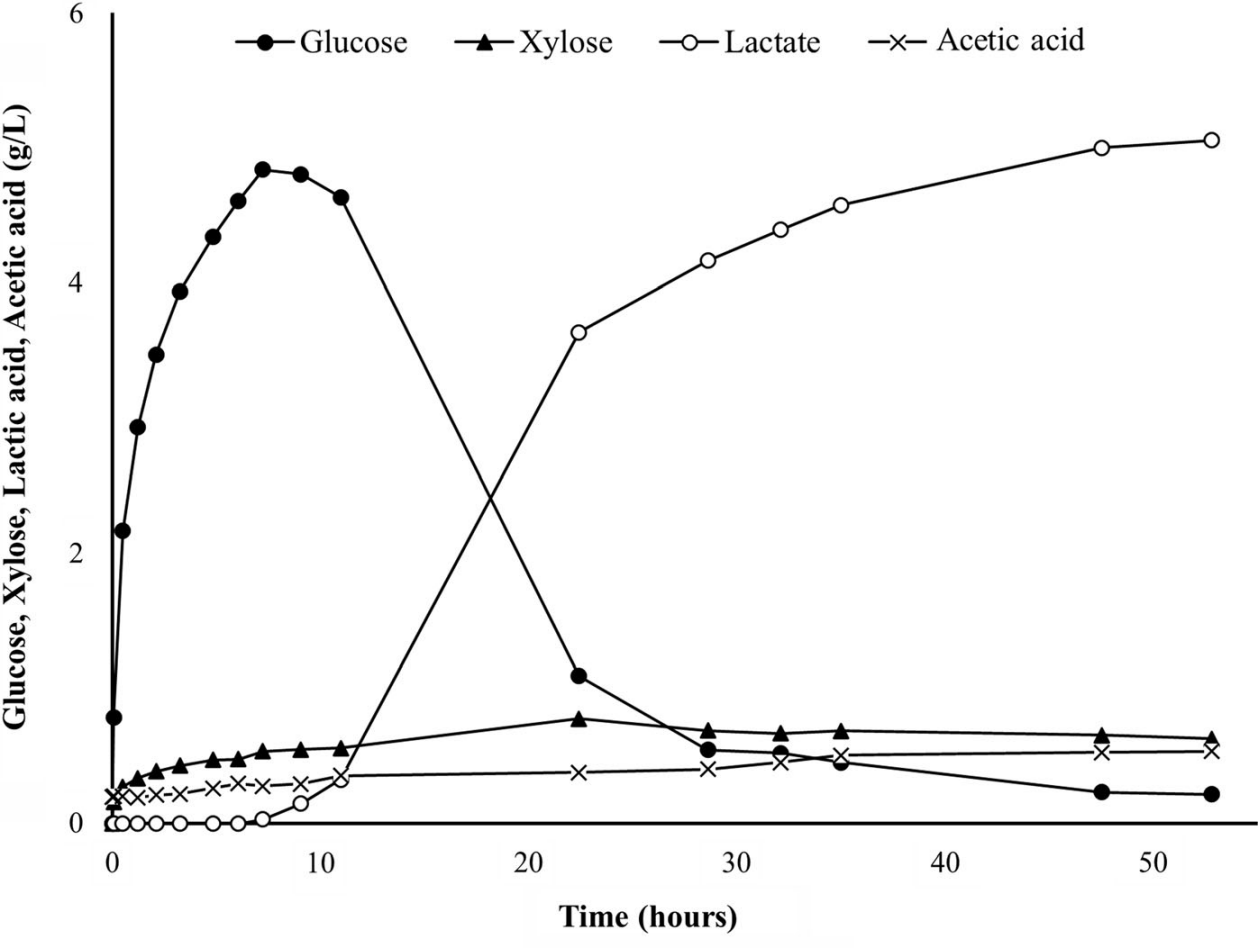
Time course of batch SSF performed with *B. smithii* SA8Eth, 2% (DW) MSW and 5 FPU/g glucan C-Tec2 at 55 °C and pH 5.0 in a 1.5 L bioreactor. Both seed culture and enzyme cocktail were added at t = 0. Culture shifted to microaerobic conditions after approximately 9 h. Glucose (solid circle), xylose (solid triangle) production/consumption, and lactate production (open circle) were tracked over 52 h

Based on the results above, *B. smithii* SA8Eth was selected for the SSF experiments. With cellulases making a significant fraction of overall SHF and SSF process costs (Tsoutsos 2010), our goal was to tailor cellulase cocktail addition to the sugar uptake requirements of *B. smithii* SA8Eth to support an initial aerobic growth phase and subsequent lactate fermentation. To achieve this, the initial rate of glucose and xylose release from 2% (w/v) MSW pulp was determined for various C-TEC2 dosages ranging from 0.625 to 10 FPU/g glucan at 55 °C and pH 5.0 (Table 1, Supplementary Fig. 3). In tandem, a fermentation time course for strain SA8Eth was carried out in a 1.5 L bioreactor at 55 °C in TSB (pH 5.0) + 20 g/L MSW derived sugars to determine the average rates of glucose consumption during aerobic growth and microaerobic fermentation, which were approximately 0.77 g/L/h (calculated between 3 and 7 h post-inoculation) and 0.37 g/L/h (calculated between 8 and 27 h post-inoculation), respectively (Fig. 2). From this, it was determined that 5 FPU/g glucan of C-TEC2—with an initial rate of glucose release for MSW pulp of 0.8 g glucose/L/h—should produce sufficient glucose to support exponential growth and fermentation of SA8Eth. Subsequently a batch SSF was performed using 2% (w/v) MSW pulp, 5 FPU/g glucan C-TEC2 and *B. smithii* SA8Eth in TSB (pH 5.0) at 55 °C. Figure 3 shows that initially the cellulase activity exceeded the capacity of the cells to take up the resulting monomers but as the fermentation progressed, this situation reversed and the monomer concentration dropped to low levels, but never to zero. Lactic acid was produced at a similar rate as when cells were grown on glucose, but unfortunately signs of lactic acid toxicity were evident and a final titre of only 5.1 g/L lactic acid was achieved after 53 h (Fig. 3), a conversion yield of 32% (w/w) on total lignocellulosic sugars. While this value is lower than the amount of lactic acid produced in 48 h at pH 7.0 (Fig. 1c), it has been reported that *B. smithii* DSM 4216 produces 58% less lactic acid when fermentation is carried out at pH 4.5 compared to pH 6.5 (Bosma et al. 2015)—which may explain the lower product yield from SA8Eth. Toxicity was also observed at a similar concentration during growth on glucose (Fig. 2), suggesting that the cessation of production was not peculiar to the growth on MSW pulp.

Although the lactic acid titres reported here do not compare with established platform producers, such as *B. coagulans* and *Lactobacillus* sp., (Aulitto et al. 2019; Prasad et al. 2020) this study serves to demonstrate that, when cellulosic SSF is being considered, other thermophilic *Bacillus* spp. and *Parageobacillus* spp. should come into consideration. While titres of 202 g/L of lactic acid have been achieved from 20% (w/v) corn starch in an SSF using *B. coagulans* WCP10-4 (Wu et al. 2014), similar titres have yet to be achieved from cellulosic substrates. Patel et al. (2006) reported production of between 10.8 and 12.6 g/L of lactic acid from three strains of *B. coagulans* using 2% (w/v) crystalline cellulose in an SSF with 5 FPU/g cellulose at 50 °C and pH 5.0. While the lactic acid tolerance of *B. smithii* SA8Eth appears to be low, there is clearly strain to strain variability and tolerance could be improved through adaptive evolution. This would then allow the use of higher solid loadings, which for the cellulosic fraction of MSW is only limited by rheology.

Recent studies have reported the isolation of cellulolytic strains of *B. coagulans* (Aulitto et al. 2017) which suggest that it may even be possible to move towards consolidated bioprocessing as an alternative to SSF. However, realistically it will always be necessary to supplement with a cellulase at the start of the process, so the ability of a given host utilize the hydrolysis products under compatible environmental conditions is the most critical factor. In addition to growing well under the temperature and pH conditions that are optimal for cellulase activity, *B. smithii* has been previously reported as being able to utilize a wide range of C5 and C6 sugars, which is an valuable characteristic considering the complex composition of lignocellulose (Bosma et al. 2016). However, while the *B. smithii* genome contains a protein annotated as a putative 6-phospho-β-glucosidase (Genbank accession No. AKP48386.1), suggesting that it has the ability to import (presumably via a PTS transporter) and internally hydrolyse cellobiose, there is no genomic evidence for the ability to utilise other short chain oligosaccharides. Conversely, this is a feature that has been observed by some *Parageobacillus* spp. (Hussein et al. 2015), a capacity which could prove to be valuable for lignocellulosic SSF as it means that the substrate need not be hydrolysed to monomeric units before utilisation. Regardless, the preliminary work done here suggests that *B. smithii* is worthy of further investigation as a candidate host for lactic acid production from lignocellulosic SSF processes.

## Conclusion

Simultaneous saccharification and fermentation for the production of commercially valuable compounds, such as lactic acid, is hindered by the fact that the operational temperature and pH of the celluloytic enzymes used for hydrolysis is incompatible with the growth and fermentation optima of most commonly used industrial microorganisms. This study expands the portfolio of thermophilic bacilli that can grow under pH and temperature conditions that are much more compatible with lignocellulosic SSF, many of which have not previously been investigated for this purpose. Further, to the best of our knowledge this is the first example in which either *B. smithii* or MSW pulp has been used for lactic acid production from SSF, which resulted in 5.1 g/L lactic acid after 53 h from 2% (w/v) pulp. This preliminary work represents the first step towards the development of a novel microbial host for lignocellulosic SSF.

## Electronic supplementary material

Below is the link to the electronic supplementary material.

Electronic supplementary material 1 (DOCX 320 kb)
