# Supplementary material for: Simultaneous saccharification and lactic acid fermentation of the cellulosic fraction of municipal solid waste using Bacillus smithii
Source: Biotechnol Lett. 2020 Nov 21;43(3):667–75. doi: 10.1007/s10529-020-03049-y (PMC7873104; doi:10.1007/s10529-020-03049-y)
Supplement: Supplementary file 1 — Electronic supplementary material 1 (DOCX 320 kb) [file 10529_2020_3049_MOESM1_ESM.docx]

***Supplementary Material***

**Simultaneous saccharification and lactic acid fermentation of the cellulosic fraction of municipal solid waste using *Bacillus smithii*.**

Micaela G. Chacón, Christopher Ibenegbu, and David J. Leak*

Department of Biology and Biochemistry, University of Bath, Bath, BA2 7AY, England

***Corresponding author:** David Leak, Department of Biology and Biochemistry, University of Bath, Bath, BA2 7AY, England, Tel: +44 (0)1225 38 4309; E-mail: m.chacon@leeds.ac.uk

**Table S1.** List of thermophilic bacterial strains used in this work, their internal reference and source/provider.

| **Internal Reference** | **Strain** | **Source/Provider** |
| --- | --- | --- |
| **00275** | *Bacillus caldolyticus* | DSMZ (natural hot pool, USA) |
| **00452** | *Bacillus caldolyticus* | Yellowstone National Park (USA) |
| **12028** | *Bacillus caldolyticus* | unknown |
| **12042** | *Bacillus caldolyticus* | [1] |
| **10020** | *Geobacillus caldoxylosylyticus* | Soil (Bracknell, UK) |
| **10087** | *Geobacillus caldoxylosylyticus* | Soil (Granville, France) |
| **10092** | *Geobacillus caldoxylosylyticus* | Soil (Granville, France) |
| **10146** | *Geobacillus caldoxylosylyticus* | Soil (Greece) |
| **SA8Eth** | *Bacillus smithii* | Silage (Woolley Farm, UK) |

**Table S2.** OD_600_ after 24 hours of growth for the nine candidate strains in TSB (pH 5.5) + 20 g/L glucose and 55℃ in flask.

| **Strain** | **OD_600_** |
| --- | --- |
| **00275** | 2.54 + 0.13 |
| **00452** | 2.71 + 0.35 |
| **10020** | 2.19 + 0.11 |
| **10087** | 2.61 + 0.29 |
| **10092** | 1.75 + 0.1 |
| **10146** | 2.26 + 0.12 |
| **12042** | 1.96 + 0.27 |
| **12028** | 2.36 + 0.18 |
| **SA8Eth** | 4.54 + 0.34 |

**Table S3.** Proportion of each product made by the nine candidate strains after 48 hours fermentation in TSB (pH 7) + 20 g/L glucose and 55℃ in tube.

| **Strain** | **Lactic acid (%)** | **Acetic acid (%)** | **Succinic acid (%)** |
| --- | --- | --- | --- |
| **00275** | 88.3 | 10.9 | 0.8 |
| **00452** | 93.5 | 5.5 | 1.0 |
| **10020** | 90.3 | 8.7 | 1.0 |
| **10087** | 63.6 | 30.4 | 6.0 |
| **10092** | 60.3 | 28.9 | 10.8 |
| **10146** | 71.2 | 22.1 | 5.8 |
| **12042** | 89.0 | 10.2 | 0.8 |
| **12028** | 88.8 | 10.8 | 0.4 |
| **SA8Eth** | 90.2 | 9.7 | 0.1 |


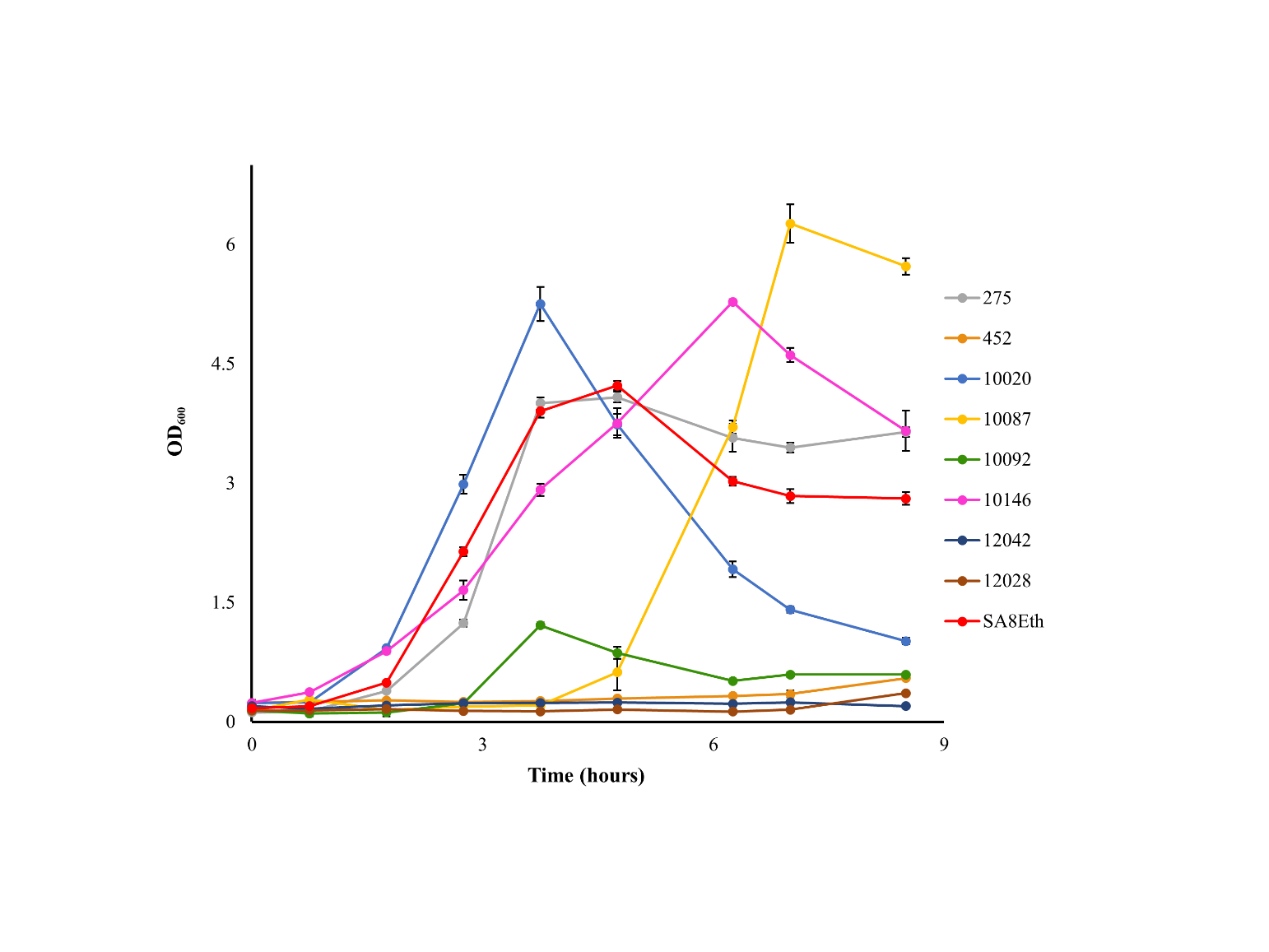


**Fig. S1** Comparison of growth (OD_600_) of the nine candidate thermophilic bacteria in TSB + 20 g/L glucose at 55°C and pH 7.0 in flask. Data are the mean + standard deviation of three biological replicates.


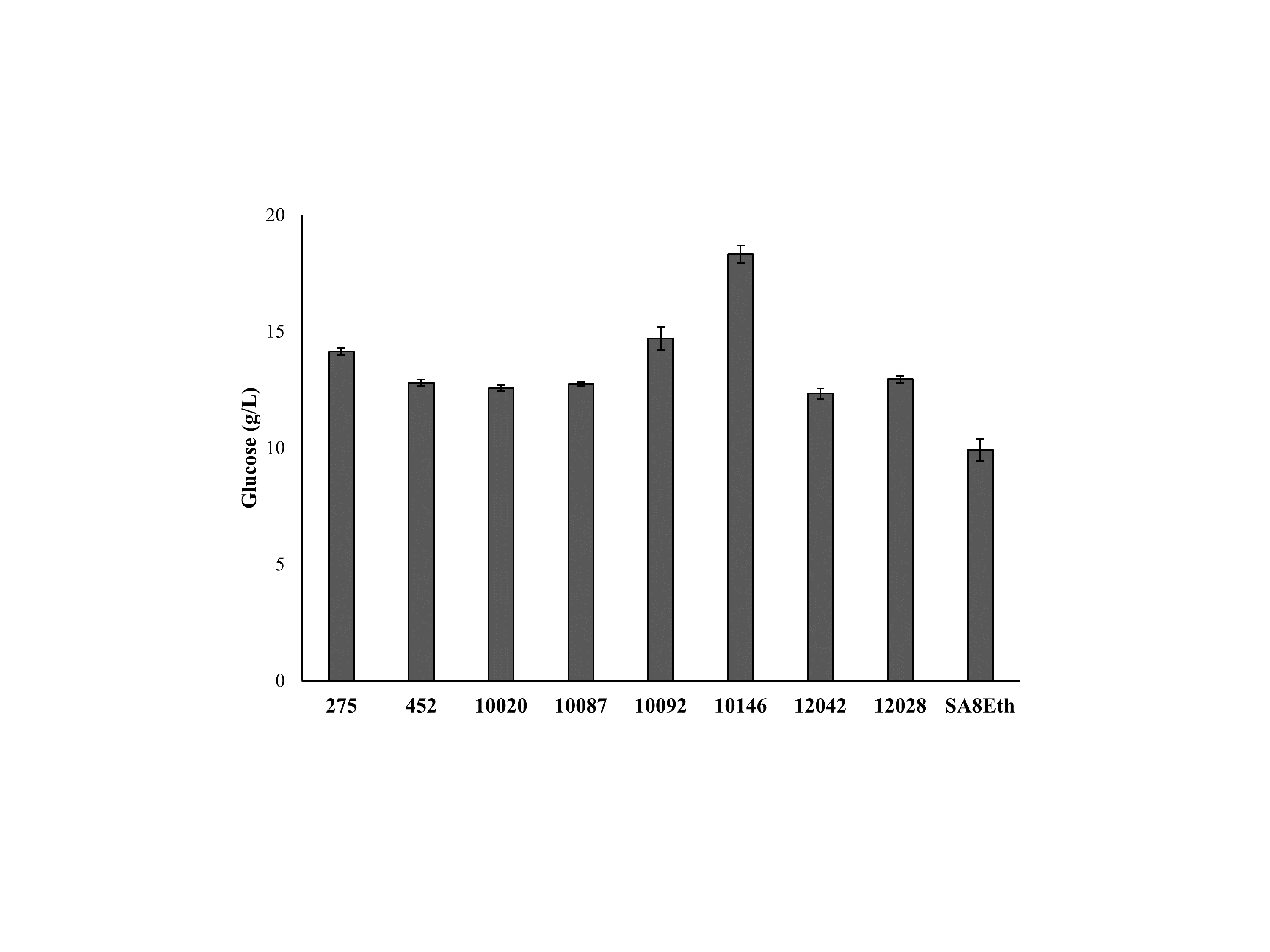


**Fig. S2** Comparison of glucose consumption from the nine candidate thermophilic bacteria in TSB + 20 g/L glucose hydrolysate sugar at 55°C and pH 5.5 after 24 hours in flask. Data are the mean + standard deviation of three biological replicates.


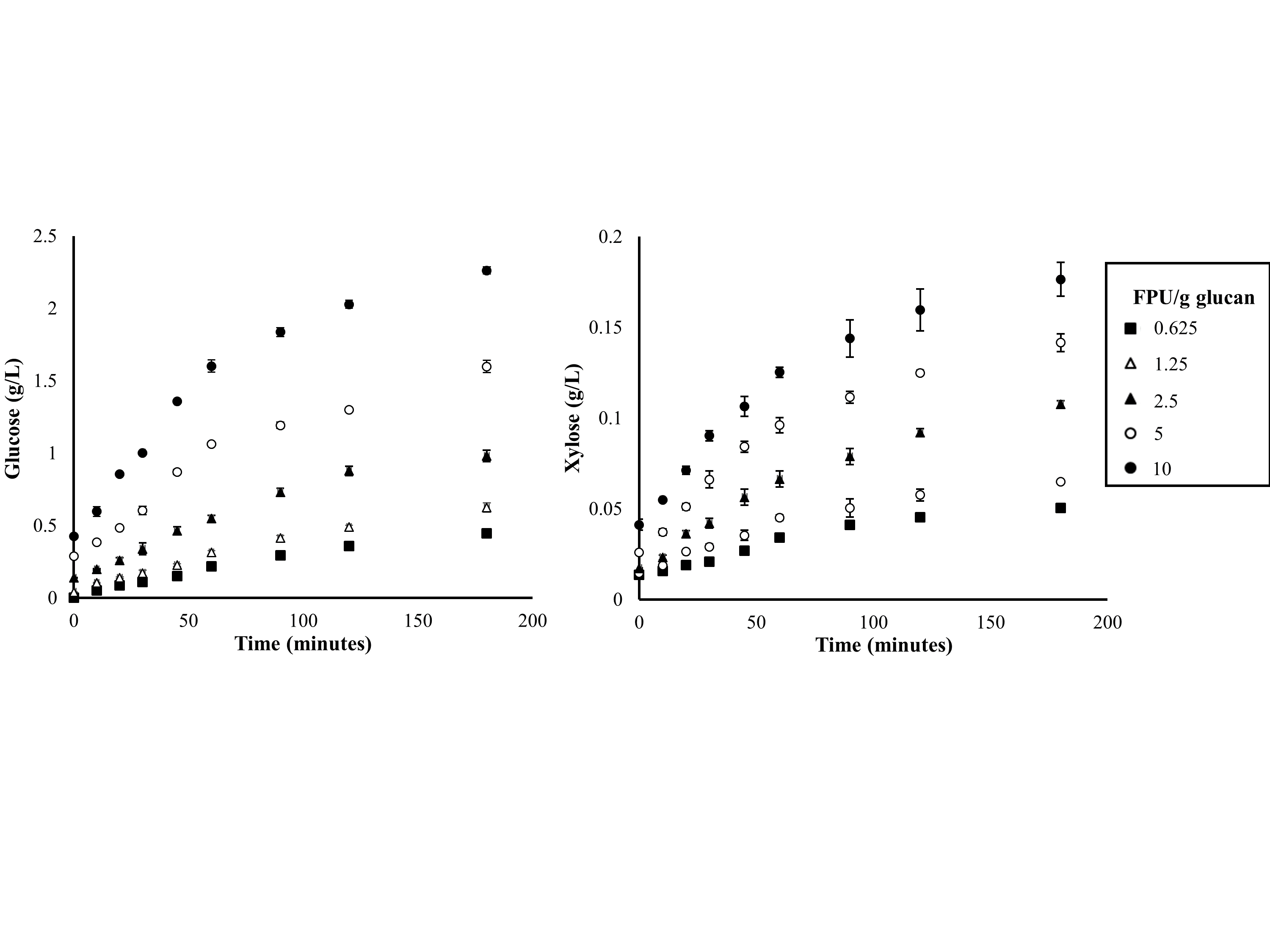


**Fig. S3** Discontinuous assay of glucose and xylose release from 2% MSW pulp (DW) and either 0.625, 1.25, 2.5, 5, or 10 FPU/g glucan of C-tec2 cellulase cocktail at 55ºC and pH 5.0 for 200 minutes. Data are the mean + standard deviation of three biological replicates.


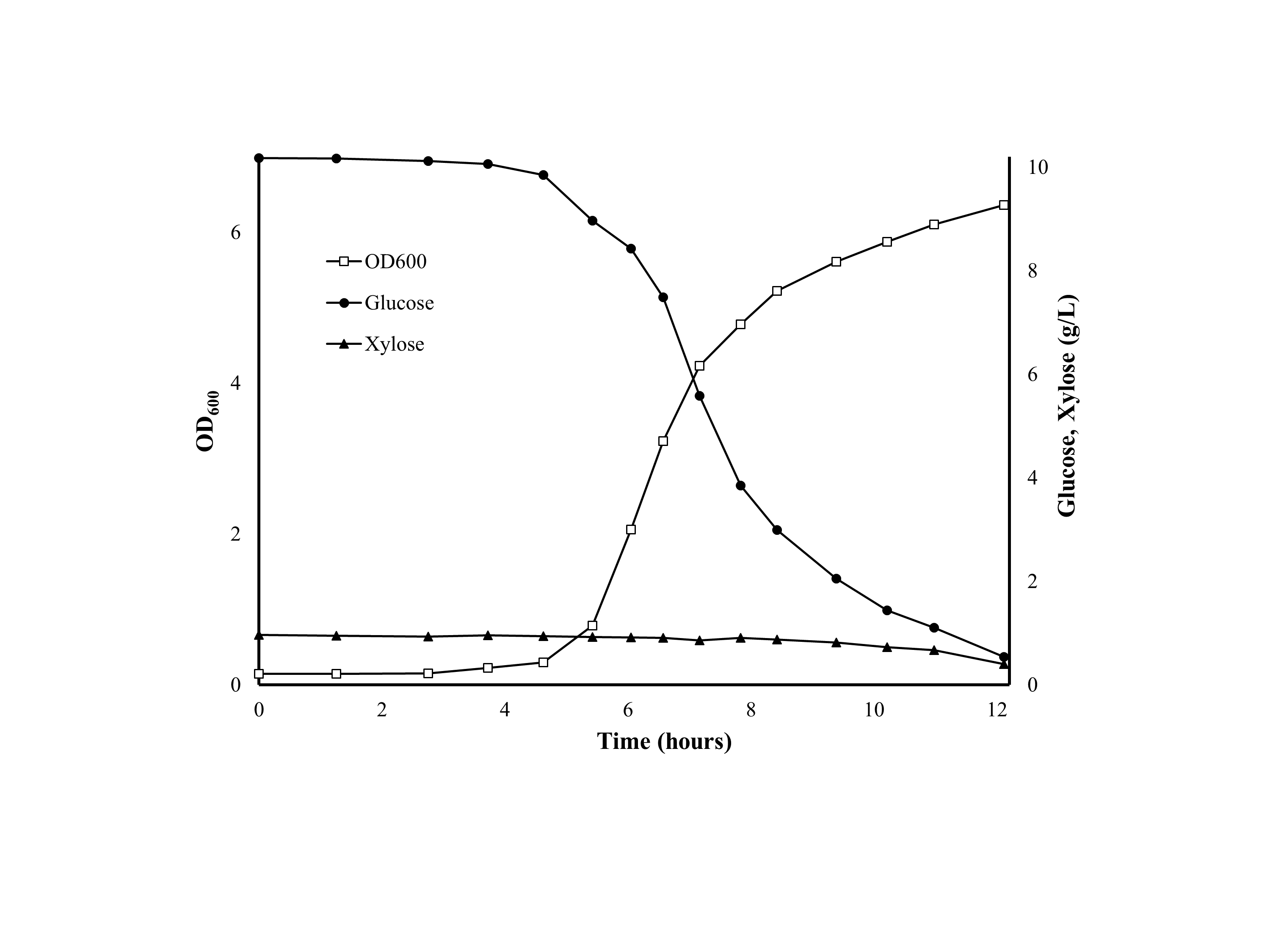


**Fig. S4** Aerobic time course of *B. smithii* SA8Eth in TSB + 10 g/L MSW hydrolysate sugar at 55ºC and pH 5.0 in a 1.5 L bioreactor. Growth (OD_600_, open square) and consumption of glucose (solid circle) and xylose (solid triangle) were tracked over 12 hours.

**References**

1. Bingham AHA, Atkinson T, Sciaky D, Roberts RJ (1978) A specific endonuclease from *Bacillus caldolyticus*. Nucleic Acids Res. https://doi.org/10.1093/nar/5.10.3457
